# Return to drug use and overdose after release from prison: a qualitative study of risk and protective factors

**DOI:** 10.1186/1940-0640-7-3

**Published:** 2012-03-15

**Authors:** Ingrid A Binswanger, Carolyn Nowels, Karen F Corsi, Jason Glanz, Jeremy Long, Robert E Booth, John F Steiner

**Affiliations:** 1 Division of General Internal Medicine and Division of Substance Dependence, University of Colorado School of Medicine, Mail Stop B180, 12631 East 17th Avenue, Aurora, CO 80045, USA; 2 Denver Health Medical Center, 501 East 28th Street, Denver, CO 80045, USA; 3 Division of General Internal Medicine, University of Colorado School of Medicine, Mail Stop B180, 12631 East 17th Avenue, Aurora, CO 80045, USA; 4 Division of Substance Dependence, University of Colorado School of Medicine, 1741 Vine Street, Denver, CO 80206, USA; 5 Institute for Health Research, Kaiser Permanente Colorado, 10065 East Harvard Avenue, #300, Denver, CO 80231, USA; 6 Department of Epidemiology, Colorado School of Public Health, 13001 East 17th Place, Mail Stop B119, Aurora, CO 80045, USA; 7 Denver Health Medical Center, 777 Bannock Street, Denver, CO 80204, USA; 8 Division of General Internal Medicine, University of Colorado School of Medicine, Mail Stop B180, 12631 East 17th Avenue, Aurora CO 80045, USA; 9 Division of Substance Dependence, University of Colorado School of Medicine, 1741 Vine Street, Denver, CO 80206, USA; 10 Institute for Health Research, Kaiser Permanente Colorado, 10065 East Harvard Avenue, #300, Denver, CO 80231, USA

**Keywords:** Drug use, Overdose, Prisoners, Relapse, Prison re-entry

## Abstract

**Background:**

Former inmates are at high risk for death from drug overdose, especially in the immediate post-release period. The purpose of the study is to understand the drug use experiences, perceptions of overdose risk, and experiences with overdose among former prisoners.

**Methods:**

This qualitative study included former prison inmates (N = 29) who were recruited within two months after their release. Interviewers conducted in-person, semi-structured interviews which explored participants' experiences and perceptions. Transcripts were analyzed utilizing a team-based method of inductive analysis.

**Results:**

The following themes emerged: 1) Relapse to drugs and alcohol occurred in a context of poor social support, medical co-morbidity and inadequate economic resources; 2) former inmates experienced ubiquitous exposure to drugs in their living environments; 3) intentional overdose was considered "a way out" given situational stressors, and accidental overdose was perceived as related to decreased tolerance; and 4) protective factors included structured drug treatment programs, spirituality/religion, community-based resources (including self-help groups), and family.

**Conclusions:**

Former inmates return to environments that strongly trigger relapse to drug use and put them at risk for overdose. Interventions to prevent overdose after release from prison may benefit from including structured treatment with gradual transition to the community, enhanced protective factors, and reductions of environmental triggers to use drugs.

## Background

Over 7.2 million people were incarcerated or on probation/parole at year-end 2009 [1]. A history of drug use or misuse is pervasive among prison inmates by every measure, including prior use, use at the time the offense is committed, drug abuse, and drug dependence [2-4]. Despite the magnitude of the problem of substance use disorders among criminal justice populations, prisoners have limited access to evidence-based substance abuse treatment during incarceration, during the transition to the community, or while under community supervision [3,5-7]. Therefore, inmates are often released without the tools to avoid returning to drugs after release from prison.

Studies in the United States and other countries have shown a high risk of drug-related death after release from prison [8-19]. Overdose rates peak in the first few weeks after release [8,20]. For instance, in prisoners released in Washington State, overdose mortality rates were 12-fold higher than what would be expected in similar demographic groups in the general population. In the first two weeks after release, the risk of overdose was even greater, with an adjusted relative risk of 129 [20]. Accidental overdoses accounted for nearly one-quarter of deaths post-release and were related to cocaine, other psychostimulants, opioids, alcohol, tricyclic antidepressants, and multiple drugs in combination. Suicide was the 4^th ^leading cause of death and likely included intentional overdoses [20].

Despite the epidemiologic data that describes a high rate of death from overdose after release from prisons, little is understood about the conditions that lead to relapse and overdose after release. Prior studies have shown that former inmates face challenges including poor housing, unemployment, psychosocial problems and barriers to health care [21-25]. We sought to better define the risk and protective factors that impact drug use and risk for overdose from the perspective of former inmates in the immediate post-release period. This information is essential to developing effective interventions that reduce risk in a real-world context. Specifically, this study was designed to better understand the drug-use experiences, perceptions, knowledge of overdose risk, and overdose experiences of former prison inmates.

## Methods

### Study design

We conducted a qualitative study using face-to-face, in-depth, semi-structured interviews with former inmates aged 18 and older who were English speaking and could understand study procedures. Former inmates who were no longer in jail but who were on current inmate status (i.e., living in the community but still considered prisoners) were excluded. We focused recruitment on former inmates within two months of release from prison to maximize recall. Interviews were scheduled as soon after recruitment as possible. The mean length of time from release to interview was 42 days (range, 5 to 82 days). The final sample included 29 inmates. Details of our interview methods with this sample have previously been described [26,27]. The study was reviewed by the Colorado Multiple Institutional Review Board. All participants reviewed a consent form prior to participating in the study.

### Study setting

Participants were initially recruited from a community health center, an urgent care center, and addiction treatment centers in the Denver, Colorado area, with subsequent snowball sampling (those who agreed to participate were asked to tell their friends and acquaintances about the study) [28]. We recruited participants by placing flyers and brochures in waiting areas and examination rooms and informing providers about eligibility criteria. Individuals who called inquiring about the study had an initial eligibility screening by phone; eligibility was confirmed in person prior to informed consent. Eligibility criteria included ability to speak English, ability to comprehend and consent to the study procedures, and age ≥ 18 years. We excluded juveniles because they have access to different post-release programs than adults. We also excluded current inmates and those whose most recent release was from jails (compared with prisons, these usually hold detainees prior to sentencing or individuals serving short sentences).

### Interviews

The interview guide was developed by the authors and included questions regarding challenges during the immediate post-release period, health-care access, HIV risk, overdose experiences, and drug use after release [26,27]. This analysis includes data from all parts of the interview, but focuses on direct questions on drug use and overdose, e.g., "After being released from prison *(this time)*, have you used any illegal drugs or prescription drugs you got on the street? *(If so,) *when was the first time? What kind of drugs did you use? What led you to use drugs since your release? How have you avoided drugs since your release? What helped you the most?" On the topic of overdose, we asked, "Some people worry about overdosing on drugs when they get out of prison, while others don't worry about this. What about you?" We also asked, "Have you had any personal experiences with overdose after release from prison or jail?" and, "Can you tell me about a time, or more than one time, when you've seen or heard about someone overdosing after their release?"

Interviews were conducted from March through June 2009. Two experienced interviewers (one male and one female) were trained in interviewing criminal-justice populations, qualitative methods, and behaviors to increase rapport and participant comfort level. Team members met periodically to debrief the interviewers.

The one-on-one interview format allowed participants to express their ideas and experiences and avoid issues of mistrust of groups [29,30]. Participants were provided $25 in the form of a check or grocery gift card. Participants who agreed to be re-contacted to verify data interpretation were compensated an additional $25 at the follow-up interview. Interviews were digitally recorded in a private setting, uploaded to a secure drive, and transcribed by a professional transcriptionist.

### Analysis

Interview transcripts were our primary data source. Transcript files reviewed for accuracy and entered into Atlas.ti^© ^qualitative data analysis software. Additional data included a brief demographic survey; interviewer summaries detailing context, process, content, and self-reflection; and notes from interviewer debriefing sessions and informant feedback.

We analyzed data using an inductive, team-based approach previously described [26,27,31-33]. Two coders reviewed the data, created primary codes, and met with the team to discuss coding until consensus was reached on a codebook. Subsequent analytic steps included an iterative team process of data collection, debriefing, and thematic analysis [29,34]. We presented the results to external groups to further refine analysis. The research team assisted with data interpretation, prioritizing salient elements, and discussing discrepancies and implications. Finally, we met with three participants to clarify key points and assess validity of our interpretations (informant feedback) [29]. The study was approved by the Colorado Multiple Institutional Review Board and obtained a Federal Certificate of Confidentiality.

## Results

Participants included 29 men (69%) and nine women (31%) with a mean age of 39 years (range, 22-57 years). Eleven participants (38%) described themselves as African American, 10 (34%) as Caucasian, 5 (17%) as Latino, and 3 (10%) as American Indian. The mean length of time since release was 42 days (range, 5-82). Over half of the participants (16/29) knew of someone who overdosed soon after release from prison, and three had personally overdosed during previous releases from prison. In the course of their interviews, 16 participants described living in shelters. The substances used by our participants included cocaine/crack, heroin, methamphetamine, marijuana, opioid-containing pain medications, benzodiazepines, alcohol, and tobacco.

### The re-entry context: social support, financial needs, and other re-entry challenges

After release, return to drug and alcohol use occurred in a context of poor social support and inadequate economic resources to support integration into the community. Social isolation was a particular problem for former inmates who were trying to stay away from drugs and alcohol. For instance, one former inmate explained:

"I just don't go around nobody. It's kind of hard 'cause my whole family gets high."

One participant described the need for social support to avoid drug use after release from prison:

"They just don't know what they're really doing to their body cause they killing themselves slowly but surely and they need somebody to help them, you know, the courage, and to tell them not to mess around with it."

Nearly all participants struggled with financial problems, and several participants described poor finances as contributing to drug use and relapse:

"Most people relapse in the first six months because it's so stressful because they have no help. There's no financial help to even get housing or to... buy clothes for work or a bus pass to even try to look for a job."

Additionally, drug trafficking in the environment where former inmates returned was considered a major problem for participants, whether they used drugs after release or not:

"With the mix of the people that have mental problems and the homeless, people that are, you know, doing drugs and it's just a mess down there [at the shelter].... They stand out there and sell drugs all day long on the corners and it's like a safe zone down there.... It's totally out of control."

In addition to the direct risk to former inmates of using drugs, many participants described the risk of violence and theft related to drug trafficking as major threats to their health and safety after release from prison. Participants perceived themselves as at substantial risk for assault and violence related to drugs and alcohol:

"The biggest threat to my safety was the area that the shelter is located in... I saw several very bad beatings. Some guy got stabbed and almost killed for a pint of vodka 'cause he had it in his pocket and the drug deals and just... it's a very dangerous area."

Overall, the multiple contextual challenges during re-entry had a strong influence on the return to drugs and alcohol after release from prison.

### Medical and mental health conditions among drug- and alcohol-involved former inmates

For some former inmates, medical and mental health needs in the re-entry context were closely linked with their drug and alcohol use disorders. Participants identified multiple comorbidities, such as diabetes, epilepsy, hypertension, chronic pain, anxiety, and depression, combined with limited access to care and medications. One woman explained that the biggest threat to her health after release was having diabetes related to alcohol use combined with poor medication continuity:

"[The biggest threat] to my health [after release]? Drinking like the way I did, 'cause I'm a diabetic and I shouldn't be drinking like that.... I almost went into a diabetic coma. My sugar was so high 'cause... the Department of Corrections didn't release me with my insulin."

Another participant with a history of oxycodone and heroin dependence described her difficulties obtaining mental-health medications as contributing to strong feelings of frustration after her release:

"The biggest threat to my health is the issue of trying to get that medication and stuff taken care of and I am really frustrated.... They'll pay for so much of your mental-health care after you get out and stuff like that, and none of that's happened yet, you know, so I'm still without a psychiatrist at this point, you know? And I have a month worth of [mental health] medicine before that runs out...."

Another participant explained the effect of frustration in the context on her drug and alcohol use:

"My biggest challenge [after release] is to not use [drugs and alcohol] and not let... all of the frustration and stuff that you feel build up...."

Later in the interview, this same participant explained that the contextual challenges she faced required that she avoid drugs and alcohol:

"The challenges that I am facing right now in my life cannot be handled without a clear mind. You know, even if [I]drink, it affects me the next day."

For the participants in this study, medical and mental-health problems interacted with poor continuity in health care, drug and alcohol use, and strong emotional reactions including frustration.

### Relapse and exposure to drugs and alcohol

Former inmates described ubiquitous exposure to alcohol, drugs, and drug trafficking in their living environments. In particular, former inmates who stayed in homeless shelters found that it took substantial effort to stay away from drugs and alcohol after release from prison. One man in his mid-forties struggling to stay abstinent from drugs after his release:

"You get asked 50 times if you want some coke before you get into the [shelter] door."

One 46-year-old man who had a place to live also described frequent exposure to drugs:

"Well, when I first got out, peoples come around asking me do you want this. Hey man, I remember you, man you used to look out for me, here, here you go. I said man, no I don't want it. I been there. I done it."

Several participants with a history of addiction described exposure to drugs as the major challenge they faced, requiring avoidant behaviors and new skills to prevent relapse. For instance, one man, whose drug involvement led to his incarceration, had successfully averted relapse since his release. He was motivated by a desire to preserve a relationship with his son, born while he was in prison. He practiced avoidant behaviors at his shelter:

"There's a lot of drug activity, kind of drug users and stuff like that at the shelter, and it's kind-of hard there.... I mean, people will walk up to me and ask me, oh, you want to buy some weed...? What are you looking for? I try and avoid those situations, those people; I said, no I'm not looking and just kind of walk away and go to a different area or something like that.... I'm really not tempted to use any drugs right now because I'm trying to get my life on a straight path but... people have offered to get me high for free, hey you want to smoke, you want to hit this pipe? You want to smoke a joint? You know, stuff like that and just avoiding it, trying to, you know, keep myself out of those situations is really the only way I've been, you know, I focus.... I think about my son, and drugs is really what took me into prison, so I don't want to use drugs 'cause they will probably take me back to prison, so I'm trying to stop myself from going in a circle."

Several participants described a return to drug use within a short period of time after release from prison. Participants described an overwhelming urge to use drugs and alcohol to cope with frustration, "numb out," and "forget about" the daily stresses of the transition period, citing easy availability combined with pressure from old friends and new acquaintances to "party." One participant explained her use of cocaine, crack, alcohol, and nonmedical benzodiazepines within one week of release as follows:

"What led me to [use] this last time... was... frustration and wanting to feel released.... [I]t was something also that I didn't go look for, that was right in the house with me, and I don't blame them for that, but it's just... I don't think I would have sought it out had it not been there."

Similarly, a young woman who had resumed intravenous methamphetamine and cocaine use after a prior release described why she relapsed:

"If you don't go to [a therapeutic community] in prison, then you never really stopped using. You just stopped intaking it, so your body still wants it, your mind still wants it, and it's all you think about while you're in prison, but if you go to rehab and people show you a different way of life, then you start thinking maybe I don't want it. But most people who are in prison are just waiting for their next hit."

For a participant who had used drugs after prior releases, the most significant challenge he faced was staying away from individuals with whom he had previously used drugs:

"[The biggest problem is] not going back to the same lifestyle that got me in prison, 'cause I have seen some of the old people that I used to hang out with, and some of them are clean and sober and doing good and some of them are still up to the same, but you know, I still care about them. They're my friends, but I just... it was hard for me to like say 'I need to go,' you know, cause I had spent so much time with them over the years that now that some of them are still getting high and still doing the things that they do, it was hard for me to just say, 'Hey, I can't not be your friend, but I just can't be around you at this time, because that's just too much of a trigger for me cause it's just one little slip up and I go back'.... [T]he hardest thing is not going back into the lifestyle that got me put in prison and finding a job."

In summary, the environments to which participants returned immediately following prison made it difficult to avoid relapse due to ubiquitous triggers to use. Despite these risks, former inmates described protective factors and responses such as strengthening family relationships, changing social networks, and avoiding former lifestyles to mitigate the risks of relapse and return to prison.

### Perceptions of overdose after release from prison

We specifically sought to learn more from former inmates about post-release overdose. Three participants had personally experienced a post-release overdose, and 16 had either witnessed or known people who had such overdoses. Most participants were aware of the dangers of post-release overdose. The reasons most frequently mentioned for overdose were the lack of knowledge about lowered tolerance levels after limited access to drugs during incarceration, the increase in potency level of street drugs over years of incarceration, and intentional overdose as a means of coping with stress and anxiety that seemed unbearable.

"The last time I OD'd, I was on parole. I did too much. I went back to my normal dosage, what I was doing before I went in and that didn't work.... I wound up in intensive care three days later from a coma.... I know that when you come out of [the Department of Corrections] your body is clean so... you need to be careful and know what you're doing... and you never know what you get."

One participant in his mid-fifties experienced fatal overdose among six of his friends and acquaintances. He described the trend of early deaths after release from prison as follows:

*"I've lost quite a few friends that have came out and were very fresh to this street life, and they OD'd on heroin you know. Just a sad thing. Of course they had only been out a couple weeks*."

Several described the overdoses of friends or acquaintances as being related to the stress of release, difficulty adhering to parole conditions, a sense of hopelessness, and a lack of ability to cope with the transition:

"It [overdose] would have to be on purpose, because parole makes it so difficult to make it."

As a result, overdose was considered by other several participants to be a mechanism for committing suicide to end such stress. One man who was interviewed five weeks after release described overdose as choosing death over going back to prison:

"It's like they purposely want you to screw up so you go back.... If I foul up, they're going to file escape charges on me... that's 48 years off the top. I'm going back for the rest of my life. I would... rather die than go back and give them 48 years of my life. So, it's like... you got a choice. Go back to prison for the rest of your life or die. They going to choose death."

Thus, overdose was considered a physiologically driven phenomena--a coping mechanism (albeit poor) for the seemingly insurmountable challenges faced by former inmates and a "way out" if the challenges became too great.

### Available services and other strategies employed by former inmates

The most commonly cited substance-abuse services available to participants after release from prison were drug and alcohol classes. Many participants also had to undergo urine toxicology screens through their case-management programs or parole offices. The parole office was perceived as structured around enforcement rather than assistance but, nonetheless, some participants obtained services and support from their parole officer. Participants also described re-entry services and community-based case management services as helpful in general. For instance, one participant identified his parole officer and Treatment Accountability for Safer Communities (TASC) case manager as helpful:

"Staying in touch with my parole and with my TASC lady, staying in touch with her."

Participants identified factors that protected them from returning to drug use, including avoidance of old neighborhoods, strong family relationships, religion and spirituality, housing, support from friends, a highly structured residential treatment program, a patient navigator, community-based organizations and programs, and self-help groups such as Alcoholics Anonymous (AA) and Narcotic Anonymous (NA). In response to a question about what has helped her avoid using drugs since release, a woman with a history of opioid dependence described the following community resources:

"[I] got involved, like, with Empowerment [a community-based organization], gone to church, been to some meetings like AA, NA, and talked to, like, my mom and stuff about it... been more open with people and not hiding it, except from my parole officer, of course (laughing), you know."

Housing away from shelters, with their associated drug use and trafficking, was of foremost importance to our participants.

"There's a lot of drugs, there's a lot of alcohol in those shelters and there should be some statutes pertaining to individuals like myself that had to parole homeless... to get housing somewhere once we're released."

One 26-year-old woman explained that a highly structured transitional residential program, paid for by the Department of Corrections, prevented her relapse.

"If I'd been in the real world, I probably would have relapsed already, but being because I am in this structured environment [residential drug treatment facility] and I have the support I need, I haven't relapsed; but if anybody is an addict and they are out there without the support, it's a probably nine-to-one chance that they're going to relapse."

Participants described learning from prior releases, which helped them through the re-entry period in their most recent release. One young woman described a long process of re-learning how she dressed and presented herself to others in order to avoid relapse after release from prison, with the assistance of a structured program:

"It's the way you act, the way you present yourself, [your] perception, you know? And it all has to be re-learned cause it's not... that you say one day, 'I want to be a dope dealer' (laughing), you know? It's something that happens with time, and everything has to be re-learned."

Despite the tremendous challenges faced by former inmates, a number of participants expressed hope about their prospects and willingness to work to maintain their sobriety and avoid relapse:

"I haven't been sober this long for a long time, so now then I'm back out and re-integrating into the community, it's kind of weird, because I didn't know how to have sober fun. I didn't know how to communicate with people without being high on drugs or drunk or... so, it's a new experience and it's kind of hard, but then at the same time, it's just... it's another challenge that I'm willing to take on."

## Discussion

Despite the high prevalence of substance use disorders among individuals in prison, our results suggest that former inmates with a history of drug use disorders and criminal drug charges are often released into environments with significant social and economic challenges, little structure, and ubiquitous drug activity. These challenges made it difficult for former inmates with drug use disorders to remain abstinent from drugs and alcohol.

Participants described a re-entry context of poor social and family support, financial insecurity, and inadequate housing. In this context, repeated triggers to use drugs in individuals who are predisposed to substance use disorders makes it difficult for them to succeed at maintaining sobriety upon re-entry. The immediate return to drug use described by some of our participants suggest that despite prolonged periods of relative abstinence in prison, the pervasive environmental stimuli experienced by former inmates with substance use disorders in the community encourages return to drug use and heightens the risk of overdose. Community-based programs, transitional housing, and other re-entry interventions should consider the need to reduce triggers for drug use to minimize the risk of relapse and overdose among former inmates (Table 1).

**Table 1:** Themes and Implications for Design of Interventions and Policies to Minimize Drug Relapse and Overdose Risk among Former Prison Inmates

Theme	Implication for Designs of Interventions and Policies
Relapse occurred in the context of poor social support, re-entry challenges and lack of financial resources.	Attention to the psychosocial and practical needs of re-entry is necessary to reduce risk.

Participants reported medical and mental-health problems combined with limited access to health care and medications.	Re-entry services should include attention to meeting basic medical needs.

Participants described ubiquitous exposure to drugs, alcohol, and drug trafficking in the environments to which they were released.	Transitional housing should be away from neighborhoods with ubiquitous drug activity. Encourage avoidant behaviors and skills to avoid exposure.

Perception of high risk of overdose after release from prison because of diminished tolerance.	Educate inmates about lower drug tolerance at release and provide bystander naloxone training and distribution.

Overdose perceived as a means of coping with unbearable stress and anxiety.	Teach new coping mechanisms for the stress and anxiety that accompanies release.

Relapse after release perceived as a coping mechanism for depression, anxiety, and frustration.	Enhance coping skills and ensure medication continuity for mental-health conditions. Motivate inmates to seek and preserve healthy relationships for support. Connect inmates with religious/spiritual institutions and community-based organizations.

Preventive factors included structured drug-treatment programs, spirituality/religion, community-based services, self-help programs, and family.	Offer inmates structured treatment for substance use disorders after release, help them link to community-based services, encourage use of self-help programs, and support positive family roles and relationships.

In addition to the effects of strong stimuli on former inmates with drug-use histories, the medical, psychosocial and economic challenges former inmates experience fuel a desire to use drugs and alcohol. Overdose was frequently perceived of as intentional, as a choice or "way out of" unbearable conditions. This suggests former inmates needed assistance to cope with the stress of transition in order to prevent intentional overdoses.

Former inmates widely described decreased tolerance as a mechanism for accidental overdose. For drugs other than opiates, biological support for this belief is lacking. Nonetheless, opioid replacement programs initiated either in prison or at release as well as distribution of antidote (e.g., naloxone) to former inmates both have the potential to prevent fatal overdoses [35,36].

In our study, former inmates identified several interventions and resources they found helpful to the maintenance of sobriety and prevention of overdose in the early post-release period. These included motivation to preserve family relationships, especially with children; support from organized religions; spirituality; housing at a distance from shelters; support from friends; highly structured residential treatment programs; community resources and organizations; and 12-step programs. Implications of our findings for the design of interventions and policies are described in Table 1.

Access to existing resources should be encouraged and supported. Highly structured programs that provide gradual re-integration of former inmates with drug use disorders into the community offer promise as a means to minimize the pervasive drug exposures they face and encourage new learned behaviors and habits. Although structured programs may require significant public investment, in light of costs associated with criminal behavior in relation to drug use and subsequent incarceration, such programs may be cost-effective. Finally, programs to reduce relapse and overdose in the early post-release period should consider addressing the economic, housing, and medical needs of former inmates to reduce the stress related to these challenges.

This study has several limitations. Qualitative data provide depth to the understanding of a problem rather than breadth [29,37]. Therefore, these results may not be generalizable to all former inmates from all correctional systems. As homelessness is common among former inmates, many of our participants lived in shelters; thus, our study may not have reflected the experiences of individuals in other housing categories. Our interviews were limited to English-speaking individuals with the capacity for scheduling and attending interviews. There may have been limited disclosure of drug use, but many of our participants were forthcoming about their drug-use histories. Although some participants had previously experienced overdoses after release and in other contexts, none of the participants had experienced an overdose during this release, which may have limited our ability to gain timely and detailed information about the circumstances which led to overdose. Finally, some of our participants generalized about their peers being released from prison; these statements may not accurately reflect the experiences of those peers.

## Conclusions

Our study offers the perspectives of former inmates on the return to drug use and overdose during the post-release period. Participants highlighted the significance of poor social support, medical problems, and inadequate financial resources to support integration into the community. Furthermore, they experienced ubiquitous exposure to drugs in the neighborhoods to which they were released. Intentional overdose was identified as "a way out" in the context of significant situational stressors, whereas accidental overdose was perceived as relating to decreased tolerance due to reduced drug exposure during incarceration. Finally, former inmates identified factors that prevented relapse and overdose, including structured drug-treatment programs, spirituality/religion, and family. These results point to several considerations for the design and implementation of interventions in the immediate post-release period.

## Competing interests

The authors declare that they have no competing interests.

## Authors' contributions

IAB, JFS, and REB conceived of the study. IAB, KFC, and CN oversaw data collection, conducted data analysis, and interpreted the data. JL facilitated data collection and contributed to data analysis and data interpretation. JG participated in interpretation of the data and reviewed drafts of the manuscript for critical content. IAB drafted the manuscript, and all authors reviewed and edited drafts of the manuscript. All authors read and approved the final manuscript.

## References

[B1] Bureau of Justice Statistics, Total Correctional Population 2011 http://bjs.ojp.usdoj.gov/index.cfm?ty=tp&tid=11

[B2] Binswanger IA Merrill JO Krueger PM White MC Booth RE Elmore JG Gender differences in chronic medical, psychiatric, and substance-dependence disorders among jail inmates Am J Public Health 2010 100 3 476 482 10.2105/AJPH.2008.149591 19696388 PMC2820077

[B3] Chandler RK Fletcher BW Volkow ND Treating drug abuse and addiction in the criminal justice system: improving public health and safety JAMA 2009 301 2 183 190 10.1001/jama.2008.976 19141766 PMC2681083

[B4] Karberg JC James DJ Bureau of Justice Statistics Special Report: Substance Dependence, Abuse, and Treatment of Jail Inmates, 2002 2005 Washington: US Department of Justice

[B5] Taxman FS Kitsantas P Availability and capacity of substance abuse programs in correctional settings: a classification and regression tree analysis Drug Alcohol Depend 2009 103 Suppl 1 S43 S53 19395204 10.1016/j.drugalcdep.2009.01.008 PMC3241974

[B6] Hammett TM Roberts C Kennedy S Health-related issues in prisoner reentry Crime & Delinquency 2001 47 3 390 409 10.1177/0011128701047003006

[B7] Taxman FS Perdoni ML Harrison LD Drug treatment services for adult offenders: the state of the state J Subst Abuse Treat 2007 32 3 239 254 10.1016/j.jsat.2006.12.019 17383549 PMC2266078

[B8] Bird SM Hutchinson SJ Male drugs-related deaths in the fortnight after release from prison: Scotland, 1996-99 Addiction 2003 98 2 185 190 10.1046/j.1360-0443.2003.00264.x 12534423

[B9] Coffey C Wolfe R Lovett AW Moran P Cini E Patton GC Predicting death in young offenders: a retrospective cohort study Med J Aust 2004 181 9 473 477 15516189 10.5694/j.1326-5377.2004.tb06402.x

[B10] Harding-Pink D Mortality following release from prison Med Sci Law 1990 30 1 12 16 2304391 10.1177/002580249003000104

[B11] Hobbs M Krazlan K Ridout S Mai Q Knuiman M Chapman R Mortality and morbidity in prisoners after release from prison in Western Australia 1995-2003 Trends Issues Crime Criminal Just 2006 320 1 6

[B12] Joukamaa M The mortality of released Finnish prisoners: a 7 year follow-up study of the WATTU project Forensic Sci Int 1998 96 1 11 19 10.1016/S0379-0738(98)00098-X 9800361

[B13] Kariminia A Law MG Butler TG Corben SP Levy MH Kaldor JM Grant L Factors associated with mortality in a cohort of Australian prisoners Eur J Epidemiol 2007 22 7 417 428 10.1007/s10654-007-9134-1 17668280

[B14] Pratt D Piper M Appleby L Webb R Shaw J Suicide in recently released prisoners: a population-based cohort study Lancet 2006 368 9530 119 123 10.1016/S0140-6736(06)69002-8 16829295

[B15] Seaman SR Brettle RP Gore SM Mortality from overdose among injecting drug users recently released from prison: database linkage study BMJ 1998 316 7129 426 428 10.1136/bmj.316.7129.426 9492665 PMC2665604

[B16] Seymour A Oliver JS Black M Drug-related deaths among recently released prisoners in the Strathclyde Region of Scotland J Forensic Sci 2000 45 3 649 654 10855971

[B17] Stewart LM Henderson CJ Hobbs MS Ridout SC Knuiman MW Risk of death in prisoners after release from jail Aust N Z J Public Health 2004 28 1 32 36 10.1111/j.1467-842X.2004.tb00629.x 15108744

[B18] Verger P Rotily M Prudhomme J Bird S High mortality rates among inmates during the year following their discharge from a French prison J Forensic Sci 2003 48 3 614 616 12762532

[B19] Merrall EL Kariminia A Binswanger IA Hobbs MS Farrell M Marsden J Hutchinson SJ Bird SM Meta-analysis of drug-related deaths soon after release from prison Addiction 2010 105 9 1545 1554 10.1111/j.1360-0443.2010.02990.x 20579009 PMC2955973

[B20] Binswanger IA Stern MF Deyo RA Heagerty PJ Cheadle A Elmore JG Koepsell TD Release from prison-a high risk of death for former inmates N Engl J Med 2007 356 2 157 165 10.1056/NEJMsa064115 17215533 PMC2836121

[B21] Iguchi MY Bell J Ramchand RN Fain T How criminal system racial disparities may translate into health disparities J Health Care Poor Underserved 2005 16 4 Suppl B 48 56 16327107 10.1353/hpu.2005.0114

[B22] Iguchi MY London JA Forge NG Hickman L Fain T Riehman K Elements of well-being affected by criminalizing the drug user Public Health Rep 2002 117 Suppl 1 S146 S150 12435838 PMC1913697

[B23] LaVigne NG Visher C Castro J Chicago Prisoners' Experiences Returning Home 2004 Washington: The Urban Institute

[B24] Fontana L Beckerman A Recently released with HIV/AIDS: primary care treatment needs and experiences J Health Care Poor Underserved 2007 18 3 699 714 10.1353/hpu.2007.0058 17675724

[B25] Mallik-Kane K Visher CA Health and Prisoner Reentry: How Physical, Mental, and Substance Abuse Conditions Shape the Process of Reintegration 2008 Washington: The Urban Institute

[B26] Binswanger IA Nowels C Corsi KF Long J Booth RE Kutner J Steiner JF "From the prison door right to the sidewalk, everything went downhill," A qualitative study of the health experiences of recently released inmates Int J Law Psychiatry 2011 34 4 249 255 10.1016/j.ijlp.2011.07.002 21802731

[B27] Adams J Nowels C Corsi K Long J Steiner JF Binswanger IA HIV risk after release from prison: a qualitative study of former inmates J Acquir Immune Defic Syndr 2011 57 5 429 434 10.1097/QAI.0b013e31821e9f41 21522015 PMC3685495

[B28] Patton M Qualitative Evaluation and Research Methods 1990 2 London: Sage 145 198

[B29] Giacomini MK Cook DJ Users' guides to the medical literature: XXIII. Qualitative research in health care A. Are the results of the study valid? JAMA 2000 284 3 357 362 10.1001/jama.284.3.357 10891968

[B30] Kenemore TK Roldan I Staying straight: lessons from ex-offenders Clin Soc Work J 2006 34 1 5 21 10.1007/s10615-005-0003-7

[B31] Thomas DR A general inductive approach for analyzing qualitative evaluation data Am J Eval 2006 27 2 237 246 10.1177/1098214005283748

[B32] Patton M Qualitative Research & Evaluation Methods 2002 3 Thousand Oaks: Sage

[B33] Ryan B Analyzing Qualitative Data: Systematic Approaches 2010 Thousand Oaks: Sage

[B34] Qualitative Research Guidelines Project 2006 http://www.qualres.org/index.html

[B35] Doe-Simkins M Walley AY Epstein A Moyer P Saved by the nose: bystander-administered intranasal naloxone hydrochloride for opioid overdose Am J Public Health 2009 99 5 788 791 10.2105/AJPH.2008.146647 19363214 PMC2667836

[B36] Tobin KE Sherman SG Beilenson P Welsh C Latkin CA Evaluation of the staying alive programme: training injection drug users to properly administer naloxone and save lives Int J Drug Policy 2009 20 2 131 136 10.1016/j.drugpo.2008.03.002 18434126

[B37] Giacomini MK Cook DJ Users' guides to the medical literature: XXIII. Qualitative research in health care B. What are the results and how do they help me care for my patients? JAMA 2000 284 4 478 482 10.1001/jama.284.4.478 10904512

